# Serotonin receptors are involved in the spinal mediation of descending facilitation of surgical incision-induced increase of Fos-like immunoreactivity in rats

**DOI:** 10.1186/1744-8069-6-17

**Published:** 2010-03-23

**Authors:** João Walter S Silveira, Quintino M Dias, Elaine A Del Bel, Wiliam A Prado

**Affiliations:** 1 Department of Pharmacology, Faculty of Medicine of Ribeirão Preto, University of Sao Paulo, Ribeirão Preto, SP 14049-900 Brazil; 2 Department of Morphology, Estomatology and Physiology, Faculty of Odontology of Ribeirão Preto, University of Sao Paulo, Ribeirão Preto, SP 14040-900 Brazil

## Abstract

**Background:**

Descending pronociceptive pathways may be implicated in states of persistent pain. Paw skin incision is a well-established postoperative pain model that causes behavioral nociceptive responses and enhanced excitability of spinal dorsal horn neurons. The number of spinal c-Fos positive neurons of rats treated intrathecally with serotonin, noradrenaline or acetylcholine antagonists where evaluated to study the descending pathways activated by a surgical paw incision.

**Results:**

The number of c-Fos positive neurons in laminae I/II ipsilateral, lamina V bilateral to the incised paw, and in lamina X significantly increased after the incision. These changes: remained unchanged in phenoxybenzamine-treated rats; were increased in the contralateral lamina V of atropine-treated rats; were inhibited in the ipsilateral lamina I/II by 5-HT_1/2B/2C _(methysergide), 5-HT_2A _(ketanserin) or 5-HT_1/2A/2C/5/6/7 _(methiothepin) receptors antagonists, in the ipsilateral lamina V by methysergide or methiothepin, in the contralateral lamina V by all the serotonergic antagonists and in the lamina X by LY 278,584, ketanserin or methiothepin.

**Conclusions:**

We conclude: (1) muscarinic cholinergic mechanisms reduce incision-induced response of spinal neurons inputs from the contralateral paw; (2) 5-HT_1/2A/2C/3 _receptors-mediate mechanisms increase the activity of descending pathways that facilitates the response of spinal neurons to noxious inputs from the contralateral paw; (3) 5-HT_1/2A/2C _and 5-HT_1/2C _receptors increases the descending facilitation mechanisms induced by incision in the ipsilateral paw; (4) 5-HT_2A/3 _receptors contribute to descending pronociceptive pathways conveyed by lamina X spinal neurons; (5) α-adrenergic receptors are unlikely to participate in the incision-induced facilitation of the spinal neurons.

## Background

Bulbospinal pathways descend to the spinal cord to either inhibit (antinociceptive) or facilitate (pronociceptive) the transmission of nociceptive inputs (for review see [1,2]). The contribution of supraspinal areas in the control of descending pronociceptive pathways was confirmed by several studies. As examples, the lesion or neural block of rostral ventromedial medulla (RVM) or periaqueductal gray (PAG) reduces the hyperalgesia induced by spinal nerve ligature [3,4], or intraplantar injection of formalin [5,6] or mustard oil [7]. Furthermore, low intensity electrical stimulation of, or low dose of glutamate into the RVM facilitates the response of spinal nociceptive neurons to noxious inputs, whereas high intensity electrical stimulation or high dose of glutamate produces the opposite effect [8].

Descending pronociceptive pathways may be implicated in states of persistent pain [9,10] and elucidation of their spinal mediation may be useful for discovery of new antihyperalgesic drugs. Spinal serotonin produces antinociception but may be pronociceptive as well (for review see [11]). Also, spinal activation of α_2_-adrenergic receptors is antinociceptive whereas activation of α_1_-adrenergic receptors is pronociceptive [12,13]. A spinal muscarinic cholinergic mechanism activated by descending noradrenergic inputs has also been proposed and it seems to be linked only with antinociception (for review see [14]).

Surgical incision of a rat paw causes primary and secondary punctate hyperalgesia [15] and increases the number of c-Fos positive neurons in the spinal cord [16], an immunohistochemical method that allows the identification of neurons activated by peripheral noxious stimulation [17]. Although being a poorly understood problem, very little effort has been dedicated toward research on the spinal mediation of descending mechanisms of post-incision pain, a model that may allow us to understand mechanisms of sensitization caused by surgery and investigate new therapies for postoperative pain.

The present study was therefore undertaken to examine the changes in the number of c-Fos positive neurons in the spinal cord of rats treated intrathecally with antagonists of serotonin, noradrenaline or acetylcholine, to evaluate whether they contribute in the spinal mediation of descending pronociceptive pathways activated by a surgical incision. The laminae I/II, V and X were systematically examined, since they are predominantly implicated in the reception, processing and rostral transmission of nociceptive information [11].

## Results

### Effects of intrathecal muscarinic cholinergic, α-adrenergic and serotonergic receptor antagonists on the number of Fos-immunoreactive neurons in the laminae I/II, and V of the rat spinal cord

**Figure 1:**
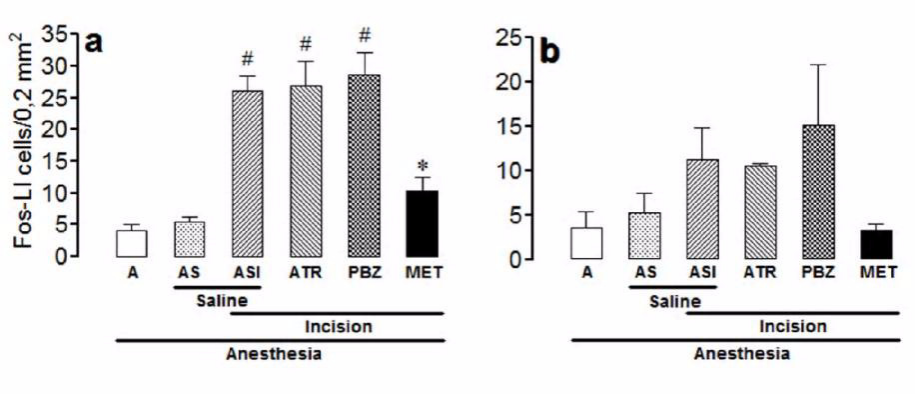
**Effects of intrathecal atropine, phenoxybenzamine or methysergide in the incision-induced Fos-like immunoreactivity in lamina I/II**. The experiments utilized 4-8 non-incised and non-catheterized anesthetized rats (A), non-incised and catheterized rats treated intrathecally with 5 μl of saline (AS), incised and catheterized rats treated intrathecally with 5 μl of saline (ASI), 30 μg/5 μl of atropine (ATR), 20 μg/5 μl of phenoxybenzamine (PBZ), or 30 μg/5 μl methysergide (MET). Surgical incision of the right hind paw was performed 3 h after PBZ or 15 min after the remaining antagonists. The number of Fos-like immunoreactive (Fos-LI) cells/0.2 mm^2 ^are shown for lamina I/II of ipsilateral (a) or contralateral (b) spinal dorsal horn. Bars are mean ± S.E.M. of the number of Fos-LI cells/0.2 mm^2 ^found in three sections taken from each rat. P < 0.05 compared to ASI (*) or AS (#).

**Figure 2:**
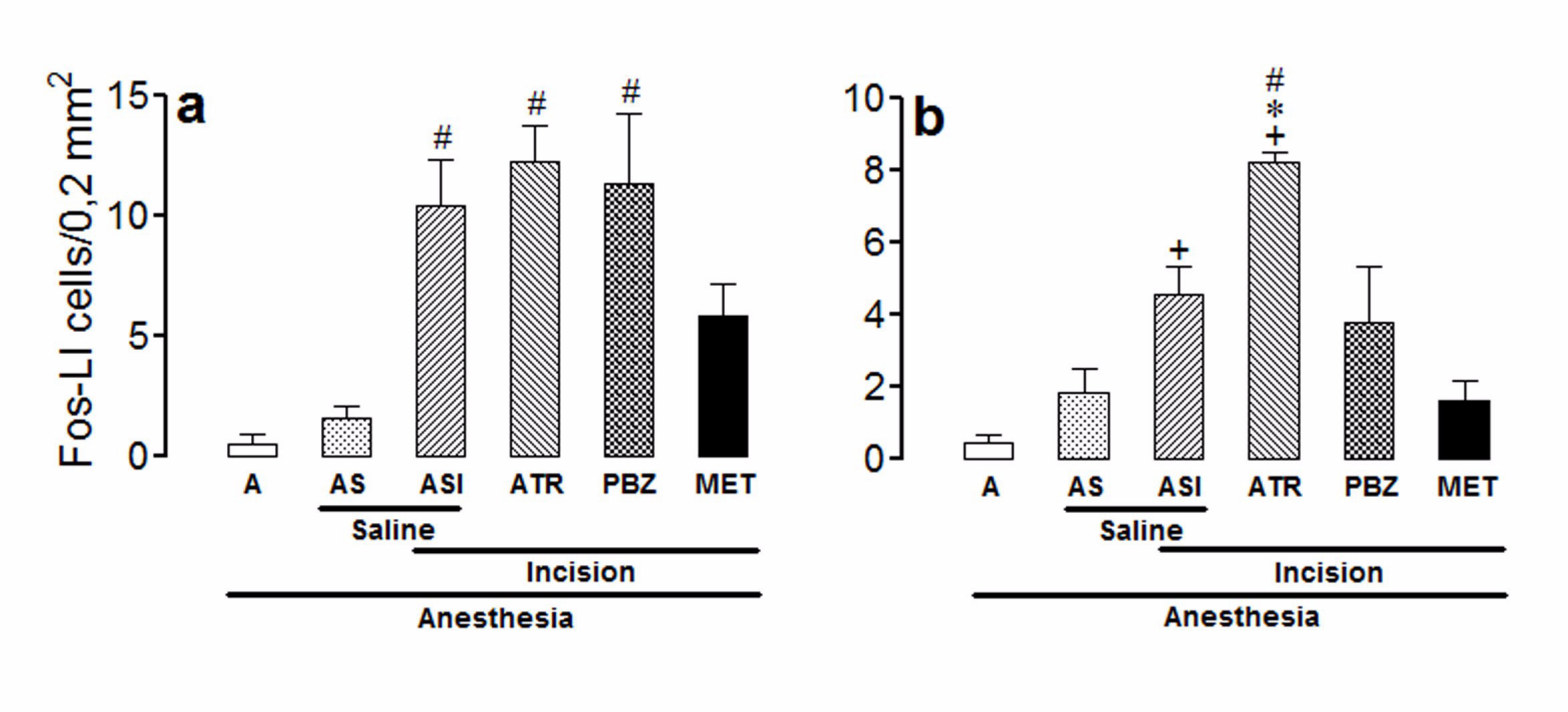
**Effects of intrathecal atropine, phenoxybenzamine or methysergide in the incision-induced Fos-like immunoreactivity in lamina V**. The experiments utilized 4-8 non-incised and non-catheterized anesthetized rats (A), non-incised and catheterized rats treated intrathecally with 5 μl of saline (AS), incised and catheterized rats treated intrathecally with 5 μl of saline (ASI), 30 μg/5 μl of atropine (ATR), 20 μg/5 μl of phenoxybenzamine (PBZ), or 30 μg/5 μl methysergide (MET). Surgical incision of the right hind paw was performed 3 h after PBZ or 15 min after the remaining antagonists. The number of Fos-like immunoreactive (Fos-LI) cells/0.2 mm^2 ^are shown for lamina V of ipsilateral (a) or contralateral (b) spinal dorsal horn. Bars are mean ± S.E.M. of the number of Fos-LI cells/0.2 mm^2 ^found in three sections taken from each rat. P < 0.05 compared to A (+), ASI (*) or AS (#).

The number of Fos-LI neurons was very low bilaterally in laminae I/II (Figure 1) and V (Figure 2) of non-incised and non-catheterized anesthetized rats (group A), and was slightly and non-significantly increased in non-incised anesthetized rats treated intrathecally with saline (group AS). The number of positive neurons was greater bilaterally in laminae I/II and V of incised rats treated intrathecally with saline (group ASI), the effect being significant at the ipsilateral laminae I/II and bilateral lamina V.

The incision-induced increase in the number of Fos-LI neurons in the ipsilateral laminae I/II of rats from the group ASI was significantly less intense following methysergide (30 μg/5 μl), and was not changed significantly by atropine (30 μg/5 μl) or phenoxybenzamine (20 μg/5 μl) (ANOVA: F_5,26 _= 24.64; P < 0.0001) (Figure 1a). Similar results were found in the contralateral laminae I/II, but the differences were not significant (ANOVA: F_5,26 _= 1.74; P > 0.05) (Figure 1b).

The incision-induced increase in the number of Fos-LI neurons in the ipsilateral lamina V of rats from the group ASI was less intense after methysergide (30 μg/5 μl), and was not changed by phenoxybenzamine (20 μg/5 μl) or atropine (30 μg/5 μl) (ANOVA: F_5,26 _= 9.94; P < 0.0001) (Figure 2a). Similar, but less intense effect occurred in the contralateral lamina V of rats treated with methysergide (30 μg/5 μl) or phenoxybenzamine (20 μg/5 μl) as compared with rats from the group ASI, but it was significantly more intense in the contralateral lamina V of rats treated with atropine (30 μg/5 μl) (ANOVA: F_5,26 _= 9.68; P < 0.0001) (Figure 2b).

**Figure 3:**
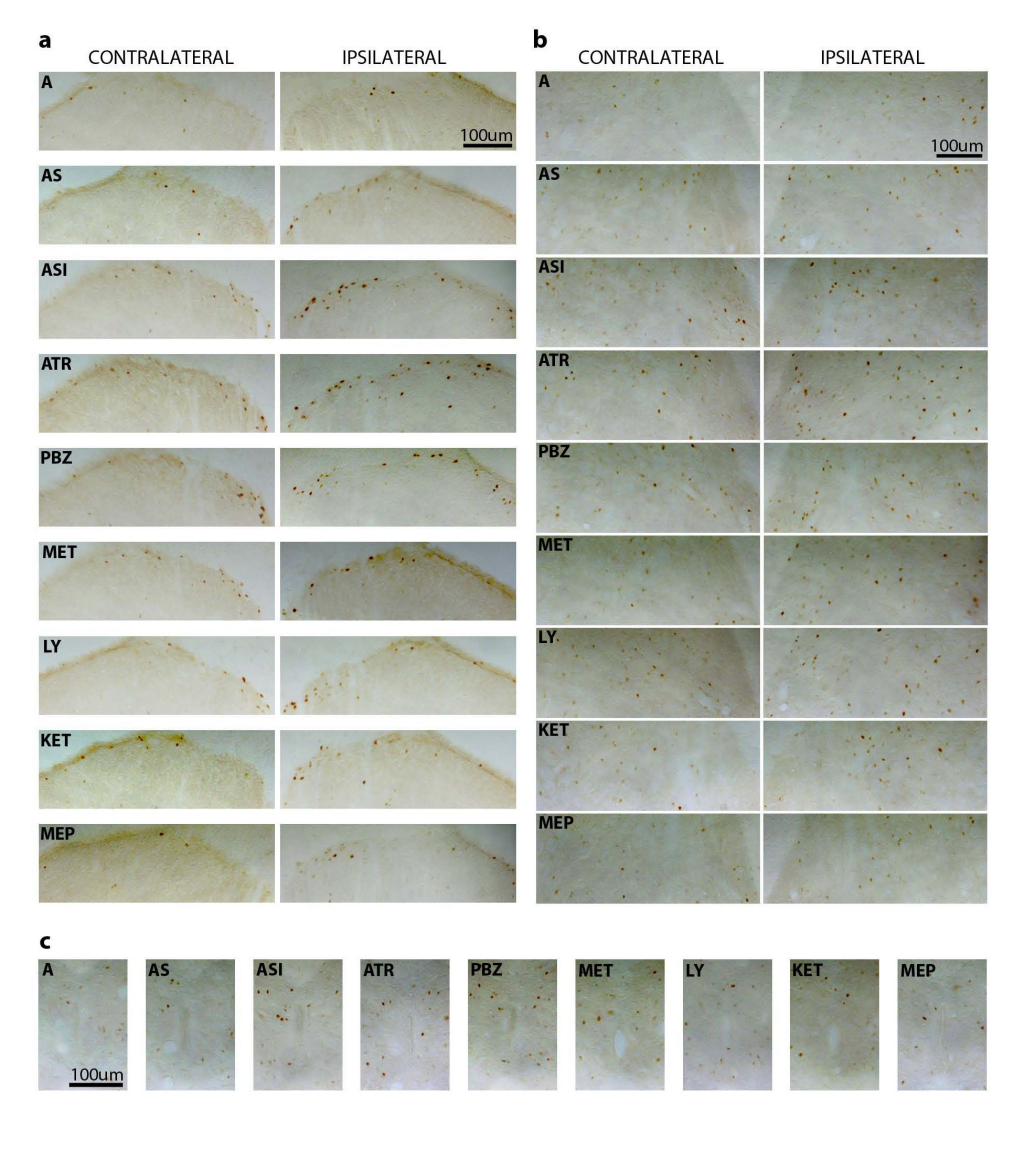
**Photomicrographs showing Fos-like immunoreactivity in rat spinal cord**. Photomicrographs were taken from 40- μm thick sections and illustrate the expression of Fos-like immunoreactivity in laminae I-II (a), V (b), and X (c) of the spinal cord gray matter (L2/L3 level), 2 h after the plantar incision of the right hind paw. of non-catheterized anesthetized rats (A), non-incised and catheterized rats treated. intrathecally with saline (AS), and incised and catheterized rats treated intrathecally with saline (ASI), atropine (ATR = 30 μg/5 μl), phenoxybenzamine (PBZ = 20 μg/5. μl), methysergide (MET = 30 μg/5 μl), LY 278,584 (LY = 100 μg/5 μl), ketanserin. (KET = 30 μg/5 μl), or methiothepin (MEP = 1.5 μg/5 μl).

Representative photomicrographs taken from sections of the spinal cords of control and test rats are given in Figure 3a and 3b for superficial and deep laminae, respectively.

### Effects of intrathecal muscarinic cholinergic, α-adrenergic and serotonergic receptor antagonists on the number of Fos-immunoreactive neurons in the lamina X of the rat spinal cord

**Figure 4:**
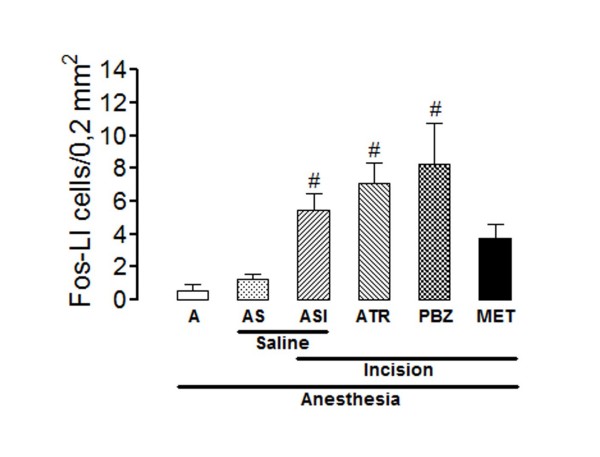
**Effects of intrathecal atropine, phenoxybenzamine or methysergide in the incision-induced Fos-like immunoreactivity in lamina X**. The experiments utilized 4-8 non-incised and non-catheterized anesthetized rats (A), non-incised and catheterized rats treated intrathecally with 5 μl of saline (AS), incised and catheterized rats treated intrathecally with 5 μl of saline (ASI), 30 μg/5 μl of atropine (ATR), 20 μg/5 μl of phenoxybenzamine (PBZ), or 30 μg/5 μl methysergide (MET). Surgical incision of the right hind paw was performed 3 h after PBZ or 15 min after the remaining antagonists. The number of Fos-like immunoreactive (Fos-LI) cells/0.2 mm^2 ^are shown for lamina X. Bars are mean ± S.E.M. of the number of Fos-LI cells/0.2 mm^2 ^found in three sections taken from each rat. P < 0.05 compared to AS (#).

The number of Fos-LI neurons in lamina X was very small in rats from group A and non-significantly higher in rats from group AS (Figure 4). The hind paw incision also induced a significant increase in the number of positive cells in lamina X, as compared to rats from group AS (ANOVA: F_5,24 _= 7.06; P < 0.0003). The hind paw incision-induced increase in the number of Fos-LI neurons in lamina X was slightly reduced by methysergide (30 μg/5 μl), and slightly increased by atropine (30 μg/5 μl) or phenoxybenzamine (20 μg/5 μl), but all changes occurred in a non significant manner. Representative photomicrographs taken from sections of the spinal cords of control and test rats are given in Figure 3c.

### Effects of intrathecal antagonists of 5-HT receptor subtypes on the number of Fos-immunoreactive neurons in the laminae I, II, and V of the rat spinal cord

**Figure 5:**
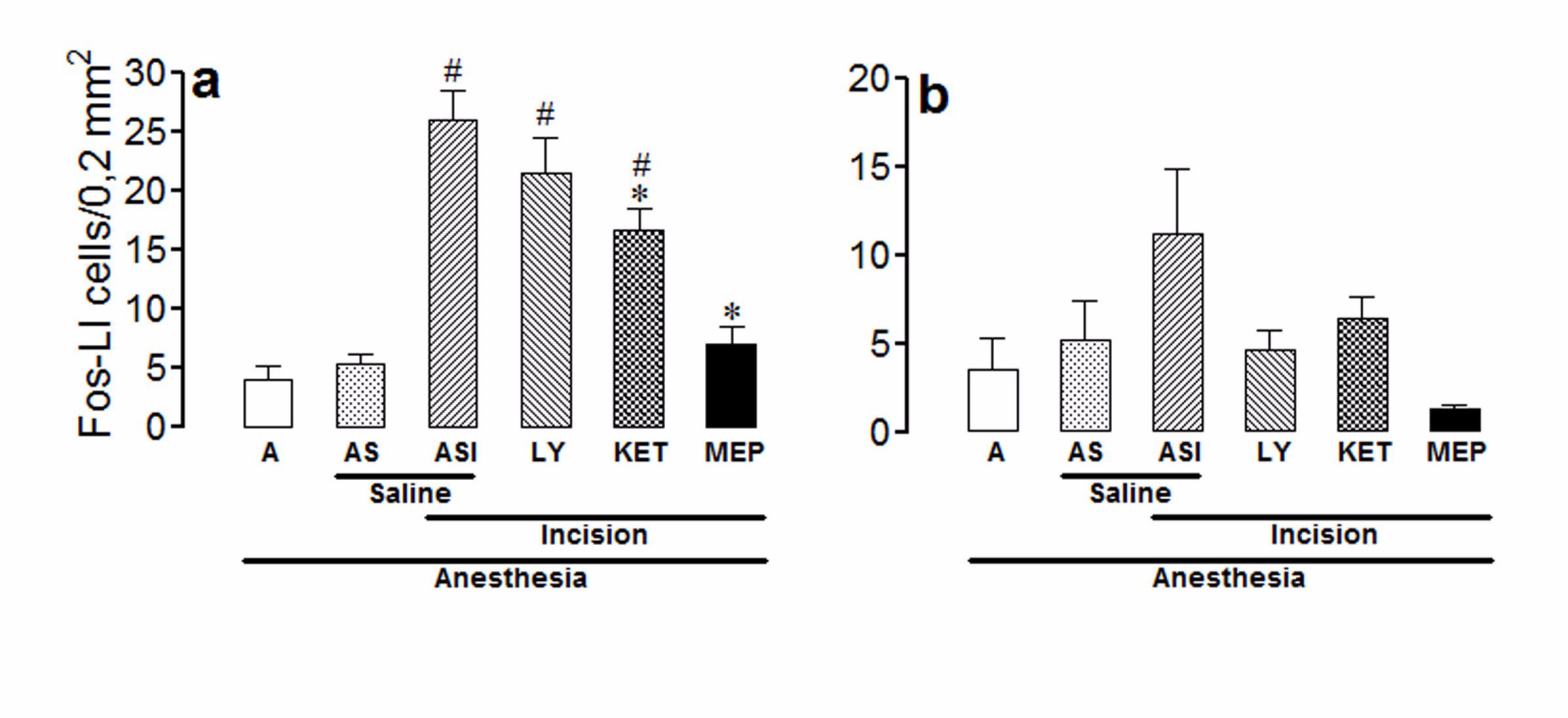
**Effects of intrathecal LY278,584, ketanserin or methiothepin in the incision-induced Fos-like immunoreactivity in lamina I/II**. The experiments utilized 4-8 non-incised and non-catheterized anesthetized rats (A), non-incised and catheterized rats treated intrathecally with 5 μl of saline (AS), incised and catheterized rats treated intrathecally with 5 μl of saline (ASI), 100 μg/5 μl of LY 278,584 (LY), 30 μg/5 μl of ketanserin (KET) or 1,5 μg/5 μl of methiothepin (MEP). Surgical incision of the right hind paw was performed 15 min after each antagonist. The number of Fos-like immunoreactive (Fos-LI) cells/0.2 mm^2 ^are shown for lamina I/II of ipsilateral (a) or contralateral (b) spinal dorsal horn. Bars are mean ± S.E.M. of the number of Fos-LI cells/0.2 mm^2 ^found in three sections taken from each rat. P < 0.05 compared to ASI (*) or AS (#).

**Figure 6:**
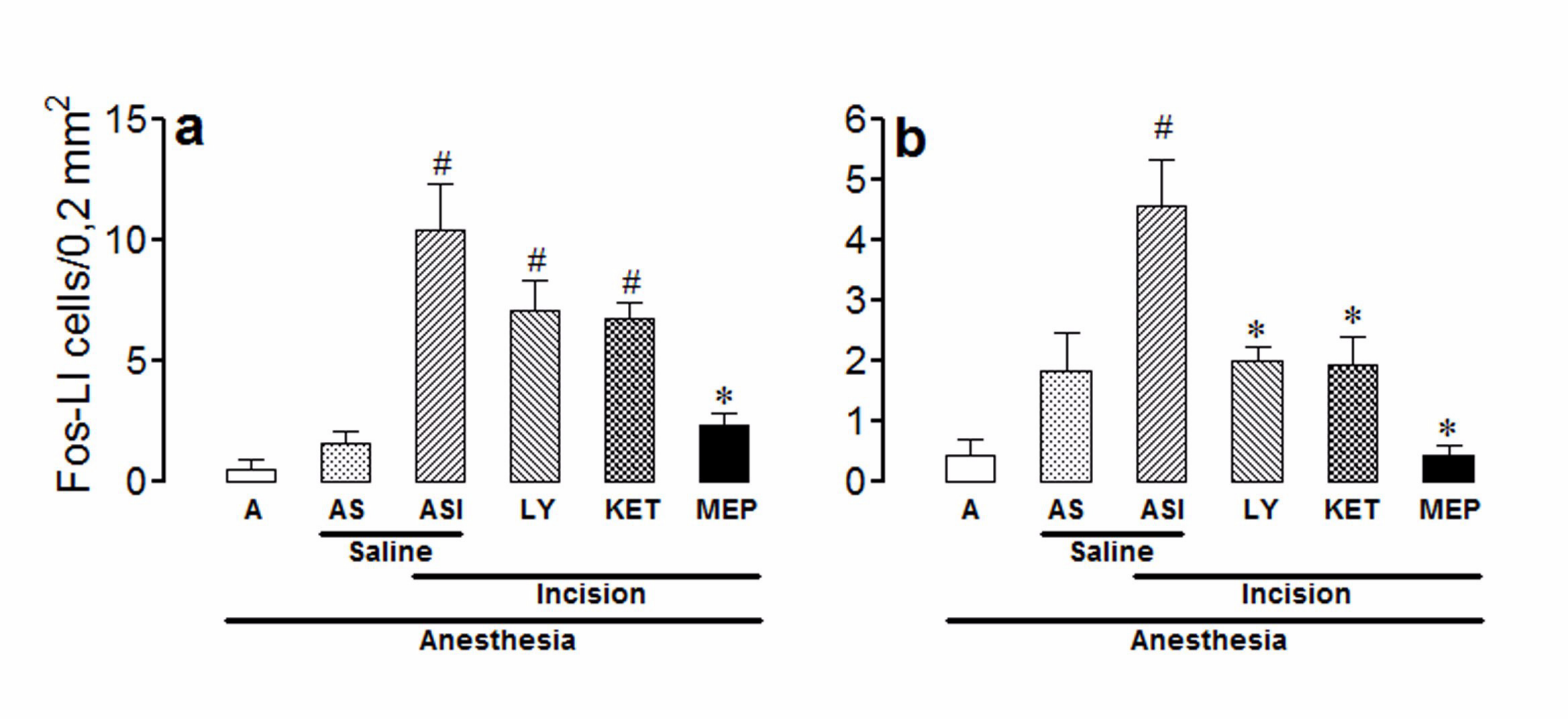
**Effects of intrathecal LY278,584, ketanserin or methiothepin in the incision-induced Fos-like immunoreactivity in lamina V**. The experiments utilized 4-8 non-incised and non-catheterized anesthetized rats (A), non-incised and catheterized rats treated intrathecally with 5 μl of saline (AS), incised and catheterized rats treated intrathecally with 5 μl of saline (ASI), 100 μg/5 μl of LY 278,584 (LY), 30 μg/5 μl of ketanserin (KET) or 1,5 μg/5 μl of methiothepin (MEP). Surgical incision of the right hind paw was performed 15 min after each antagonist. The number of Fos-like immunoreactive (Fos-LI) cells/0.2 mm^2 ^is shown for lamina V of ipsilateral (a) or contralateral (b) spinal dorsal horn. Bars are mean ± S.E.M. of the number of Fos-LI cells/0.2 mm^2 ^found in three sections taken from each rat. P < 0.05 compared to ASI (*) or AS (#).

The number of Fos-LI neurons was very low in laminae I/II (Figure 5) and V (Figure 6) of rats from group A, and was slightly but non-significantly increased in rats from group AS. The number of positive neurons was higher bilaterally in laminae I/II and V of incised rats treated with saline (group ASI), the effect being significant at the ipsilateral laminae I/II and bilateral lamina V.

The incision-induced increase in the number of Fos-LI neurons in the ipsilateral laminae I/II was significantly reduced by methiothepin (1,5 μg/5 μl) or ketanserin (30 μg/5 μl), but was not changed by LY 278,584 (100 μg/5 μl) (ANOVA: F_5,30 _= 24.77; P < 0.0001) (Figure 5a). However, no significant difference was demonstrated in the contralateral laminae I and II among the experimental groups (ANOVA: F_5,30 _= 2.02; P > 0.05) (Figure 5b).

The incision-induced increase in the number of Fos-LI neurons in the ipsilateral lamina V was also reduced by methiothepin (1,5 μg/5 μl), ketanserin (30 μg/5 μl) or LY 278,584 (100 μg/5 μl), but only the effect of methiothepin was significant (ANOVA: F_5,26 _= 9.94; P < 0.0001) (Figure 6a). In contrast, ketanserin, LY 278,584 and mainly methiothepin all reduced significantly the incision-induced increase in the number of positive cells in the contralateral lamina V (ANOVA: F_5,30 _= 7.27; P = 0.0001) (Figure 6b).

Representative photomicrographs taken from sections of the spinal cords of control and test rats are given in Figure 3a and 3b for superficial and deep laminae, respectively.

### Effects of intrathecal antagonists of 5-HT receptor subtypes on the number of Fos-immunoreactive neurons in the lamina X of the rat spinal cord

**Figure 7:**
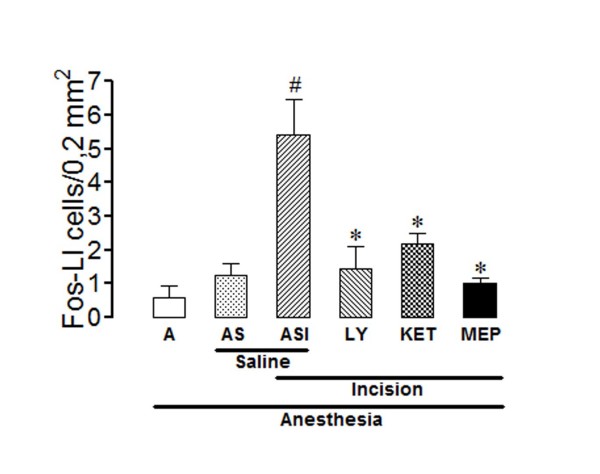
**Effects of intrathecal LY278,584, ketanserin or methiothepin in the incision-induced Fos-like immunoreactivity in lamina X**. The experiments utilized 4-8 non-incised and non-catheterized anesthetized rats (A), non-incised and catheterized rats treated intrathecally with 5 μl of saline (AS), incised and catheterized rats treated intrathecally with 5 μl of saline (ASI), 100 μg/5 μl of LY 278,584 (LY), 30 μg/5 μl of ketanserin (KET) or 1,5 μg/5 μl of methiothepin (MEP). The number of Fos-like immunoreactive (Fos-LI) cells/0.2 mm^2 ^are shown for lamina X. Surgical incision of the right hind paw was performed 15 min after each antagonist. Bars are mean ± S.E.M. of the number of Fos-LI cells/0.2 mm^2 ^found in three sections taken from each rat. P < 0.05 compared to ASI (*) or AS (#).

The number of Fos-LI neurons in lamina X was very small in rats from group A and was non-significantly superior in rats from group AS (Figure 7). The hind paw incision also induced a significant increase in the number of positive cells in lamina X, as compared to rats from group AS, the effect being significantly reduced by LY 278,584 (100 μg/5 μl), ketanserin (30 μg/5 μl) or methiothepin (1,5 μg/5 μl) (ANOVA: F_5,28 _= 9.26; P < 0.0001). Representative photomicrographs taken from sections of the spinal cords of control and test rats are given in Figure 3c.

## Discussion

Fos protein is a product of the proto-oncogene c-Fos, which is expressed in the neuronal nuclei few hours after an appropriate stimulus [18]. The increase in the expression of Fos-LI neurons in spinal cord has been used as a marker of the neuronal activity induced by noxious stimuli in various pain models [17]; for review see [19,20]. Although being a poorly understood problem, very little effort has been dedicated toward research on the spinal mediation of descending mechanisms of post-incision pain, a model that may allow us to understand mechanisms of sensitization caused by surgery and investigate new therapies for postoperative pain.

The present study confirmed that a surgical incision in a rat hind paw increased significantly the number of Fos-LI neurons in the spinal dorsal horn laminae I/II ipsilateral, and lamina V bilateral to the incised paw as shown elsewhere [16,21,22].

Also, a significant increase in the number of Fos-LI neurons were also found in the lamina X of the spinal gray matter following the incision, as described elsewhere subsequent to peripheral or visceral nociceptive inputs [23].

Incision of a hind paw increases both the response of spinal wide dynamic range cells to mechanical stimulation [15] and the spontaneous activity of nociceptive primary afferents [24]. The model of incision pain differs from inflammatory pain models [25,26], but the tissue reactions to incision are likely to involve some inflammatory responses to local injury [27].

Evidence that descending influences can affect spinal phenomena induced by a paw incision includes the demonstration that incision pain is further increased in rats with lesion of dorsolateral funiculus [28], that conveys descending pain inhibitory pathways [11], or electrolytic lesion of the anterior pretectal nucleus [29], that participates in the activation of descending mechanisms of pain control [30]; in the ipsilateral spinal cord, the incision-induced increase in the number of Fos-LI neurons was significantly reduced in the superficial lamina and significantly increased in the deep lamina of animals previously treated with bupivacaine in the contralateral anterior pretectal nucleus [16]; finally, descending pathways are known as the only sources of serotonin in the spinal cord [2,11,31].

Acetylcholine, noradrenaline and serotonin modulate noxious inputs processing in the spinal cord (for review see [11]. Atropine, a non selective muscarinic receptor antagonist was used here at 30 μg/rat, 3 fold superior to the dose known to be effective against the neostigmine-induced antinociception in rats [32].

Phenoxybenzamine, a non selective α-adrenergic receptor antagonist was used here at 20 μg/rat, 2.5 fold superior to the dose early shown to be effective against glutamate-induced analgesia from the RVM of rats [33]. Nonetheless, neither the atropine nor the phenoxybenzamine changed the incision-induced increase in the number of Fos-LI neurons in the laminae I/II, bilaterally, ipsilateral lamina V, or in lamina X. Consequently, muscarinic and α-adrenergic receptors are unlikely to participate in mechanisms that facilitate the response of spinal neurons to noxious inputs evoked by the incision. On the contrary, the increase in the number of Fos-LI neurons in the lamina V contralateral to the incised paw was significantly greater in atropine- than in saline-treated rats. In agreement to earlier studies [34-36], we interpret the result as indirect evidence that a muscarinic cholinergic mechanism responds to a surgical incision reducing the response of spinal neurons to noxious inputs from the contralateral paw. A possibility remains that the depression of c-Fos labeling is due to toxic effects of the antagonists used in the study. However, as far as we know, there is no report showing that at the doses used in the study, the intrathecal antagonists induces toxic effects against spinal dorsal horn cells in rats.

Among the seven classes of serotonin receptors (5-HT_1-7_) currently known (for review see [37]), at least three (5-HT_1/2/3/_) were found in the spinal cord [38,39] and are implicated in spinal pain processing [40-43]. The antinociceptive effect of 5-HT may also occur via interaction with spinal 5-HT_4 _receptors [44,45], but experiments involving pharmacological blockade of these receptors were not conducted in this study.

The increase in the number of Fos-LI neurons in the lamina I/II ipsilateral to the incised paw was significantly inhibited by antagonists for 5-HT_1/2B/2C _(methysergide), 5-HT_2A _(ketanserin) or 5-HT_1/2A/2C/5/6/7 _(methiothepin) receptors, but was not changed by a 5-HT_3 _antagonist (LY 278,584). The same effect was also significantly inhibited in the lamina V ipsilateral to the incised paw by methysergide or methiothepin, but remained unchanged following ketanserin or LY 278,584. Therefore, 5-HT_1/2A/2C _receptors in laminae I/II and 5-HT_1/2C_receptors in lamina V, but not 5-HT_3 _receptors in either laminae I/II or V, are involved in the activity of a descending pathway that facilitates the response of spinal neurons to noxious inputs induced by a surgical incision in the ipsilateral paw.

The significant increase in the number of Fos-LI neurons in the contralateral lamina V was inhibited by all the serotonergic antagonists used in the study. Thus, 5-HT_1/2A/2C/3 _receptors contribute to the effect of serotonin in the activity of a descending pathway that facilitates the response of spinal neurons to noxious inputs induced by a surgical incision in the contralateral paw. Finally, the increase in the number of Fos-LI neurons in lamina X was significantly inhibited by LY 278,584, ketanserin or methiothepin, but not by methysergide. We then conclude that 5-HT_2A/3 _receptors contribute to the effects of serotonin in a descending pronociceptive pathway conveyed by lamina X spinal neurons.

Altogether, these results support the involvement of serotonin in descending mechanisms that facilitate the response of spinal neurons to nociceptive inputs in the spinal cord. Serotonergic nerve terminals found in the spinal cord originate from supraspinal sources [2,11,31] and, therefore, descending pathways utilizing serotonin somehow excite nociceptive cells in the spinal cord while a postoperative pain is in course. Bulbospinal influences from the RVM contribute to facilitation of noxious inputs and development of secondary hyperalgesia in persistent inflammatory, neuropathic, and visceral pain models[46]. Primary and secondary hyperalgesia observed after a surgical incision do not appear to be modulated by descending influences from the RVM, thus supporting the view that incision pain involves different mechanisms compared with inflammatory and neuropathic pain[47]. However, in this study Pogatzki *et al*., utilized rats 5 days after RVM lesion and, therefore, it is possible that a structure located more rostrally assumes the control of the primary and secondary hyperalgesia observed after a surgical incision. In fact, several other studies have shown the involvement of descending pain pathways in this model [29,30,48-51].

The spinal actions of serotonin has long been associated to suppression of the responses to nociceptive inputs [52-54], but evidence has accumulated questioning whether descending serotonergic pathways play an exclusive spinal suppressive effect against nociceptive inputs [53,55]. The activation of spinal serotonin receptors has been associated with both pronociceptive and antinociceptive effects depending on algesimetric test, drug dosage, duration of the treatment and pathophysiological condition [56-58].

The spinal dorsal horn contains high concentrations of 5-HT_1A_, and 5-HT_1B _receptors [39,59], but the occurrence of 5-HT_1D _receptors is possible [60,61]. The activation of spinal 5-HT_1A _may result analgesia [62,63] (see also [64]), or hyperalgesia [65,66], while the activation of spinal 5-HT_1B _receptors produces antinociception [67,68].

The presence of spinal 5-HT_2A _[69,70], and 5-HT_2C _receptors [71,72] has already been demonstrated. Stimulation of spinal 5-HT_2A _receptors is pronociceptive [53,73], but the presence of 5-HT_2A _receptors on spinal inhibitory interneurons supports an antinociceptive role for 5-HT [74,75]. The activation of spinal 5-HT_2C _receptors excites neurons [55,76,77], and its distribution in the spinal cord is compatible with a pronociceptive role of 5-HT in the dorsal horn [12,78]. Conversely, 5-HT_2C _sites on spinal inhibitory interneurons allow a potential antinociceptive role for serotonin [79].

Studies have demonstrated that 5-HT_3 _receptors are concentrated in superficial layers of the dorsal horn [59,80] and a significant proportion is located on the terminals of C fibres [12,81]. The activation of the 5-HT_3 _receptors depolarizes neurons in dorsal root ganglions and, therefore, is expected to increase the transmitter release from the primary afferent terminals into the spinal cord (see [64]). In line with the present results, the activation of spinal 5-HT_3 _receptors increases nociception [62,82,83], and blockade of spinal 5-HT_3 _receptor reduces the hypersensitivity of spinal dorsal horn neurons of nerve-ligated rats [84]. A pronociceptive role for 5-HT_3 _receptors in the spinal cord following activation of descending pronociceptive pathways has already been proposed [84,85].

It is also noteworthy, that a direct facilitation by serotonin of glutamatergic synapses has been demonstrated in the spinal cord [86,87] and, therefore, this mechanism may account for some of the excitatory effects of serotonin found here.

There is evidence that a peripheral damage simultaneously triggers both descending inhibition and facilitation onto both primary and secondary spinal hyperalgesic mechanisms, but the balance for primary hyperalgesia is different from the balance for secondary hyperalgesia (for review see [48]).

Behavioral nociceptive responses of our animals were not evaluated during this study but it may be assumed that the increased number of Fos-LI neurons in the dorsal horn ipsilateral to the incised paw essentially reflects primary hyperalgesia. By extension, a secondary hyperalgesia evoked by central sensitization may be assumed to occur when the effect was observed in the contralateral dorsal horn (see [88]). Therefore, our results show that incision-induced primary and secondary hyperalgesias seem to be spinally mediated by 5-HT_1/2A/2C _and 5-HT_1/2A/2C/3 _receptors, respectively.

Few studies have accessed which neurotransmitters are involved in the spinal processing of hyperalgesia in the mode of post-incision pain. They have shown that noradrenergic receptors are involved in the spinal mediation of descending inhibitory pathways in the primary hyperalgesia of postoperative pain [29,49,51]. Few reports are also available regarding secondary hyperalgesia after post-incision pain [47,89-94] but, as far we know, none of them have studied spinal neurotransmitters involved in descending mechanisms.

## Conclusions

In conclusion (Table 1), 5-HT_1/2A/2C _receptors in laminae I/II and 5-HT_1/2C _receptors in lamina V contribute to the effects of serotonin in descending pathway that facilitates the response of spinal neurons to noxious inputs from the ipsilateral paw (primary hyperalgesia?); 5-HT_1/2A/2C/3 _receptors mediate the effects of a descending pathway that facilitates the response of spinal neurons to noxious inputs from the contralateral paw (secondary hyperalgesia?); and 5-HT_2A/3 _receptors contribute to the effects of serotonin in a descending pronociceptive pathway conveyed to lamina X spinal neurons. Finally, the study confirms that spinal muscarinic cholinergic mechanism responds to a surgical incision reducing the response of spinal neurons to noxious inputs from the contralateral paw.

**Table 1:** Effects of antagonists in the incision-induced increase of Fos-like immunoreactivity in the rat spinal cord

Antagonists	LI/LII		LV		LX
		
	ipsi	contra	ipsi	contra	
Atropine (muscarinic)	-	-	-	↑*	-
Phenoxybenzamine (α-adrenergic)	-	-	-	-	-
Methysergide (5-HT_1/2B/2C_)	↓*	-	↓#	↓#	↓#
LY 278,584 (5-HT_3_)	-	-	-	↓*	↓*
Ketanserin (5-HT_2A_)	↓*	-	-	↓*	↓*
Methiothepin (5-HT_1/2A/2C/5/6/7_)	↓*	-	↓*	↓*	↓*

Antagonists were injected intrathecally. Abbreviations: ipsi = ipsilateral and contra = contralateral to the incised hind paw; ↓ = decrease and ↑ = increase of the effect produced by the incision on the Fos-like immunoreactivity. (*) Significantly different from intrathecal saline-treated incised rats. (#) Abolished the significance of the incision-induced increase of Fos-like immunoreactivity but did not differ significantly from intrathecal saline-treated incised rats.

## Methods

### Subjects

Male Wistar rats (200-250 g) were used in this study. Animals were housed two to a cage under controlled temperature (22 ± 1°C) and on a 12-h light-dark cycle, with the dark cycle beginning at 07:00 h, and had free access to food and water. The experiments were approved by the Commission of Ethics in Animal Research, Faculty of Medicine of Ribeirão Preto, University of São Paulo (Number 009/2004). The guidelines of the Committee for Research and Ethical Issues of IASP [95] were followed throughout the experiments.

### Surgery

Each rat was anesthetized with halothane via a loose-fitting, cone-shaped mask, and catheterization of the spinal subarachnoid space was performed as described elsewhere [96]. Briefly, a 20-gauge Weiss needle was introduced through the skin into the L5-L6 intervertebral space. The correct positioning of the needle was assured by a typical flick of the tail or hind paw. A 12-mm length of polyethylene tubing (PE tubing, o.d. = 0.4 mm, dead space = 10 μl) was then introduced through the needle to protrude 2.0 cm into the subarachnoid space in a cranial direction. The needle was then carefully removed and the tubing anchored to the back skin with a cotton thread suture. Drug or saline was injected intrathecally soon after the catheterization in a volume of 5 μl over a period of 60 s, followed by 5 μl of sterile saline at the same rate to flush the catheter. The plantar side of the right hind paw was prepared with a 10% povidone-iodine solution 15-min later. A 1-cm longitudinal incision was made with a surgical blade, through the skin and fascia of the plantar region, starting 0.5 cm from the proximal edge of the heel, as described elsewhere [97]. The plantaris muscle was elevated, but its origin and insertion were left intact. After hemostasia, the skin was apposed with one single suture of 5-0 nylon, and the animal was allowed to recover in the home cage for a period of 2 h. The positioning of the intrathecal catheter was verified when the spinal cord was removed for Fos immunohistochemistry.

### Fos immunohistochemistry

The animals were sacrificed with an intraperitoneal overdose of sodium thiopental performed 2 h after the plantar incision, and perfused transcardially with saline followed by 4% paraformaldehyde in 0.1 M PBS, pH 7.4. The spinal cord was removed, fixed for 2 h in paraformaldehyde and stored for at least 48 h in 30% sucrose. The side ipsilateral to the incised paw was marked with a little knife cut. The samples were then frozen in Tissue Teck (Sakura^®^). Fos immunohistochemistry was processed, as described elsewhere [98], on 40 μm transverse sections obtained with a cryostat (Leica CM 1850) from L2-L3 spinal cord segments. The tissue sections were successively washed and incubated for 1 h in goat anti-rabbit biotinylated antibody (1:400 in PBS; Vector Laboratories, Burlingame, CA). They were then processed by the avidin-biotin immunoperoxidase method (Vectastain ABC kit, Vector Lab, Burlingame, CA, U.S.A.), and then Fos-like immunoreactivity (FLI) was revealed by the addition of chromogen diaminobenzidin (Sigma).

All reactions were performed at room temperature. Fos-like immunoreactivity was quantified using an image analysis system (Leica, Quantimet 500, Leica Microsystems Inc. Cambridge, UK) that identified and counted immunostained neurons according to a gray level that was empirically determined prior to analysis. The number of Fos-like immunoreactive (Fos-LI) neurons/section was calculated as the mean of the three sections examined for each rat. For analysis of the laminar distribution of Fos-LI neurons, the spinal cord gray matter was divided into three regions (laminae I-II, V, and X). Assessment of Fos-like immunoreactivity was conducted in a blind manner.

The number of Fos-LI neurons per region on both the ipsilateral and contralateral slides was counted in fixed area sizes (0.2 mm^2 ^for spinal cord laminae I/II, V or X), using a software 9.0 image analysis system (W. Rasband, National Institute of Health). Only rats showing catheter tip positioned at the dorsal spinal cord were considered for data analysis.

### Drugs

Atropine sulfate, a non selective muscarinic cholinergic antagonist, phenoxybenzamine hydrochloride, a non selective α-receptor antagonist, methysergide, a 5-HT_1/2B/2C _antagonist [99], ketanserin, a 5-HT_2A _antagonist [100], methiothepin, a 5-HT_1/2A/2C/5/6/7 _antagonist [101], and LY 278,584, a 5-HT_3 _antagonist [102,103], were purchased from Sigma (St Louis, MO, USA) and diluted in sterile isotonic saline at the moment of the injection.

### Experimental design

All animals were anesthetized and allocated to one of four experimental groups. A group for the overall control of the experiment had non-incised and non-catheterized anesthetized rats (group A), which were sacrificed 2 h after the beginning of anesthesia. A group for control of the effects of intrathecal catheterization had non-incised rats treated intrathecally with saline (5 μl) and were sacrificed 2 h later (group AS). A group for control of the effects of the hind paw incision had rats treated intrathecally with saline and submitted to the surgical incision of the right hind paw performed 15-min later (group ASI). Test groups had rats submitted to paw incision carried out 3 h after intrathecal phenoxybenzamine or 15-min after methysergide, atropine, LY 278,584, ketanserin or methiothepin. Animals from group ASI and test groups were sacrificed 2 h after the incision.

### Statistics

The mean (± SEM) of the number Fos-LI cells/0.2 mm^2 ^of 3 sections from the spinal cord of 4 - 8 animals per group was taken to allow comparisons among the different treatments. Comparisons of groups were made using one-way ANOVA followed by the Tukey's Multiple Comparison test. The level of significance was set at P < 0.05 in all cases.

## Competing interests

The authors declare that they have no competing interests.

## Authors' contributions

JWSS performed a large portion of the experiments and analyzed data; QMD performed a portion of experiments; EAD helped with the experimental design and analysis of Fos immunohistochemistry photographs; WAP supervised the study and helped with the writing of the manuscript. All authors read and approved the final manuscript.
